# MOS-FET as a Current Sensor in Power Electronics Converters

**DOI:** 10.3390/s150818061

**Published:** 2015-07-24

**Authors:** Rok Pajer, Miro Milanovič, Branko Premzel, Miran Rodič

**Affiliations:** 1 Faculty of Electrical engineering and computer sciences, University of Maribor, Smetanova 17, SI-2000 Maribor, Slovenia; E-Mails: rok1pajer@gmail.com (R.P.); miro.milanovic@um.si (M.M.); 2 Piktronik, Cesta k Tamu 17, SI-2000 Maribor, Slovenia; E-Mail: branko@piktronik.com

**Keywords:** power electronics, converter, MOS-FET, current measurement, thermal model

## Abstract

This paper presents a current sensing principle appropriate for use in power electronics’ converters. This current measurement principle has been developed for metal oxide semiconductor field effect transistor (MOS-FET) and is based on $U_{DS}$ voltage measurement. In practice, shunt resistors and Hall effect sensors are usually used for these purposes, but the presented principle has many advantages. There is no need for additional circuit elements within high current paths, causing parasitic inductances and increased production complexity. The temperature dependence of MOS-FETs conductive resistance $R_{DS-ON}$ is considered in order to achieve the appropriate measurement accuracy. The “MOS-FET sensor” is also accompanied by a signal acquisition electronics circuit with an appropriate frequency bandwidth. The obtained analogue signal is therefore interposed to an A-D converter for further data acquisition. In order to achieve sufficient accuracy, a temperature compensation and appropriate approximation is used ($R_{DS-ON}=R_{DS-ON}\left(\vartheta_{j}\right)$). The MOS-FET sensor is calibrated according to a reference sensor based on the Hall-effect principle. The program algorithm is executed on 32-bit ARM M4 MCU, STM32F407.

## 1. Introduction

Recently, there has been significant interest in any kind of power electronics’ converters for using current sensors for current control purposes [1,2,3]. The more frequently-used current measurement principles in power electronics’ converter applications are based on the magnetic principle and/or voltage measurement on a shunt resistor. Both of them must also be equipped with appropriate analogue signal acquisition electronic circuits. However, high currents, flowing through shunt resistors, causes additional power losses. On the other hand, the shunt measurement principle reduces the costs of power electronic converters, whilst those sensors based on the magnetic principle are relatively expensive. Based on the shunt resistor principle, MOS-FETs (basic elements in power converters) can be used as current sensors. MOS-FETs are appropriate as sensors in many different application, as was investigatedin [4,5,6]. The on-state resistance of MOS-FETs, $R_{DS-ON}$, can act as the shunt resistance and can be used as it. The benefit of such organized measurement devices is obvious: there is no need for additional elements for current measurement purposes in power converters.

Motivations for this research effort have included cost savings associated with reducing the number of current sensors. Some of the basic challenges associated with achieving effective and accurate current measurement are dealing with temperature compensation, linearization and the calibrating of a whole measurement chain [7,8]. The use of MOS-FETs as a current sensor is considered in [9], where the authors describe an analog solution to be used for transistor protection. Furthermore, in [10], the authors applied the MOS-FET as a sensor, placing attention on the temperature circuit, as well, but an additional MOS-FET was used as a sensor circuit. The synchronization issues must also be considered when using the micro-controller. Such an organized sensor has limited accuracy, depending on the quality of the sensor’s instrumental amplifier and the evaluation of the $R_{DS-ON}$ changes. The sensor circuit has limited bandwidth and disturbance rejection. $R_{DS-ON}$ depends on gate-source voltage $U_{GS}$ and MOS-FET junction temperature. The range of resistance changes due to the temperature is significant, which means that the MOS-FET sensor’s accuracy strongly depends on junction temperature at a particular time instant. The MOS-FET junction temperature $\left(\vartheta_{j}\right)$ is impossible to measure; therefore, the thermal model is used for evaluation of the $R_{DS-ON}=R_{DS-ON}\left(\vartheta_{j}\right)$. This phenomena was studied in [11,12].

This paper proposes an algorithm for using the MOS-FET resistance $R_{DS-ON}$ for current measurement purposes in a power converter circuit. The Introduction is followed by Section 2, where the measuring principle is presented considering the estimation of the MOS-FET junction temperature by evaluating of the MOS-FET power dissipation. Furthermore, an appropriate acquisition electronics circuit is featured for $U_{DS}$, case and heat sink temperature measurement. Section 3 describes the experimental setup. Experimental results and the discussion are given in Section 4. Section 5 concludes the paper.

## 2. The Measuring Principle

**Figure 1:**
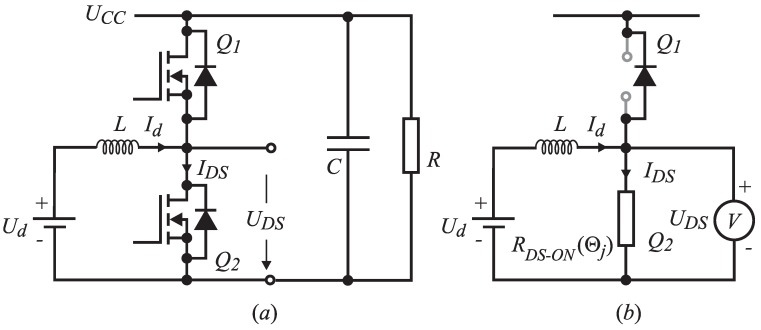
The step-up converter scheme.

**Figure 2:**
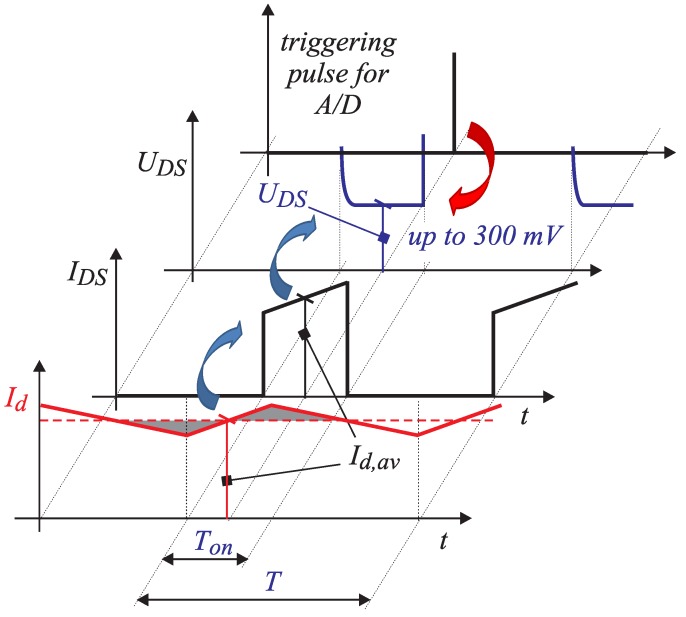
Synchronization of the $U_{DS}$ measurement.

Voltage $U_{DS}$ is measured when the MOS-FET is in the on-state. Figure 1a shows the half-leg circuit with the appropriate inductor as a DC-DC step-up converter. Voltage over $Q_{2}$ terminals (drain source) appears as a consequence of the current flowing through transistor $Q_{2}$ on-state resistance $R_{DS-ON}$, as indicated in Figure 1b. Figure 2 shows the expected waveforms of inductor current $I_{d}$, MOS-FET current $I_{DS}$ and the voltage on the MOS-FET $U_{DS}$. The upper MOS-FET $Q_{1}$ is in the off-state and the lower $Q_{2}$ in the on-state. When digital control is applied, the current sensor is usually triggered in the middle of the time interval $T_{on}$. Due to measurement synchronization, the measured signal corresponds to the average value of the inductor current at the interval $T_{s}$ indicated by $I_{d,av}$. If the resistance $R_{DS-ON}$ is known by measuring the $U_{DS}$ in the middle of interval $T_{on}$, by applying Ohm’s law, the average current can be calculated as follows:

(1) $$I_{d,av}=\frac{U_{DS}}{R_{DS-ON}}$$

**Figure 3:**
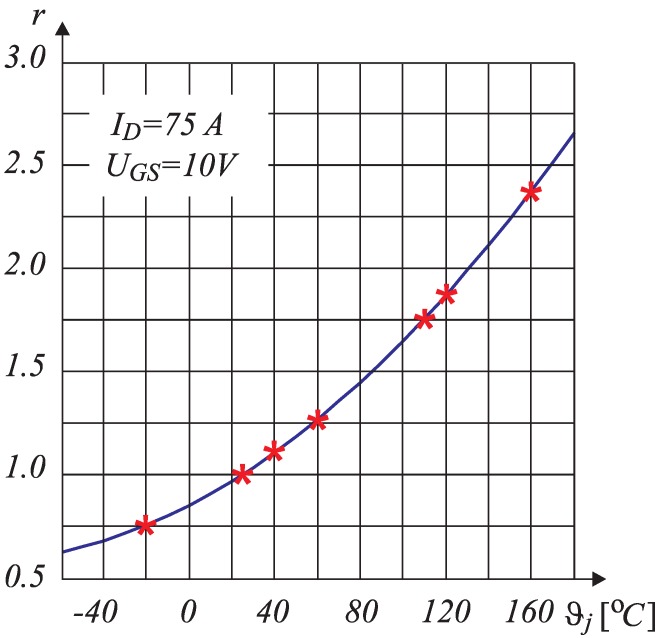
Resistance $R_{DS-ON}$ as a function of junction temperature $\vartheta_{j}$.

In general, the $R_{DS-ON}$ is known, but it is not constant. In order to determine the $R_{DS-ON}$ accurately, it is necessary to evaluate its dependence on the junction temperature $\vartheta_{j}$. The data-sheet [13] of the chosen MOS-FET transistor IRFB4110 was used for the evaluation of the function $R_{DS-ON}=R_{DS-ON}\left(\vartheta_{j}\right)$. The temperature dependence was evaluated by the curve shown in Figure 3. Those points indicated by * were extracted from the curve and are arranged in Table 1, where *r* represents the normalized MOS-FET resistance. For the chosen MOS-FET:

(2) $$r=R_{DS-ON}\left(\vartheta_{j}\right)/R_{DS-ON}\left(25{}^{o}C\right)$$

where $R_{DS-ON}\left(25{}^{o}C\right)=3.7\;m\text{Ω}$. As suggested in [14] and by using the data indicated in Table 1, the approximated analytical term for *r* is obtained by a second order quadratic equation with coefficients $K_{0}$, $K_{1}$ and $K_{2}$ as follows:

(3) $$r=K_{0}\vartheta_{j}^{2}+K_{1}\vartheta_{j}+K_{2}$$

where $K_{0}$, $K_{1}$ and $K_{2}$ are indicated in Table 2. As follows from the above analyses, the crucial issue for appropriate estimation of on-state resistance $R_{DS-ON}$ is evaluation of the junction temperature $\vartheta_{j}$, because the value of $R_{DS-ON}$ can change by 300% according to the $\vartheta_{j}$.

**Table 1:** Points extracted from the data-sheet.

$\vartheta_{j}$ [${}^{\circ }$C]	−20	25	40	60	110	120	160
*r*	0.750	1.000	1.120	1.250	1.750	1.875	2.375
$R_{DS-ON}\left(\vartheta_{j}\right)\left[m\text{Ω}\right]$	2.775	3.700	4.144	4.625	6.675	6.938	8.788

**Table 2:** Temperature characteristic’s coefficients.

Coefficients	$K_{0}$	$K_{1}$	$K_{2}$
	$2.61\times 10^{-5}$	$5.36\times 10^{-3}$	$8.49\times 10^{-1}$

### 2.1. Estimation of Junction Temperature

Unfortunately, the MOS-FET junction temperature is impossible to measure directly and can only be evaluated by using the MOS-FET’s thermal model and by the calculated power dissipation. In general, the MOS-FET power dissipation $P_{d}$ consists of two main parts as follows:

(4) $$P_{d}=P_{con}+P_{sw}$$

where $P_{con}$ represents the conductive and $P_{sw}$ represents the switching losses. MOS-FET power dissipation presents the power sources with an equivalent thermal circuit, which causes $\vartheta_{j}$ to increase, as well asits influences on $R_{DS-ON}$.

**Figure 4:**
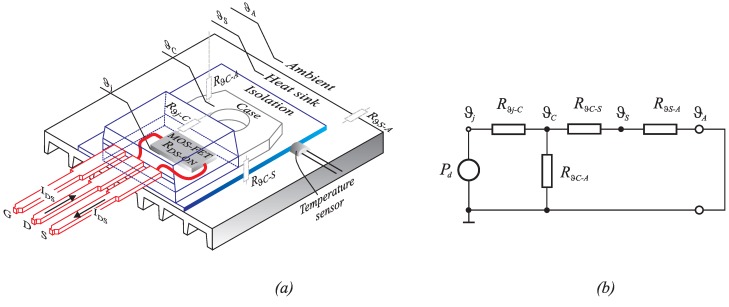
The MOS-FET TO-220 package; (**a**) thermal components inside the TO-220 package; (**b**) equivalent thermal circuit.

**Figure 5:**
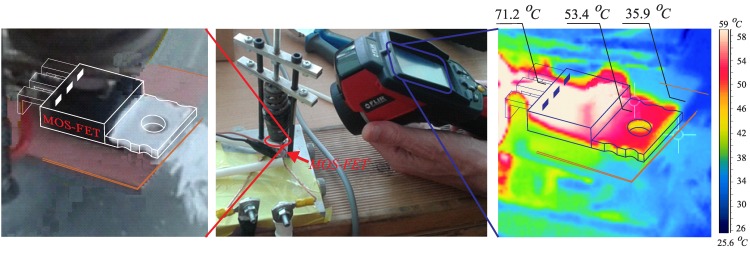
Measurement of $R_{\vartheta j-S}$ for the MOS-FET TO-220 package.

The nearest point, where temperature measuring can be performed, is the transistor case or heat sink, as indicated in Figure 4a. This can be estimated by using the transistor’s simple thermal equivalent circuit, as shown in Figure 4b. Due to the thermal resistances, the junction temperature $\vartheta_{j}$ is higher than the case temperature $\vartheta_{C}$, and $\vartheta_{C}$ is higher than the heat sink temperature $\vartheta_{S}$. The junction and case temperatures can be calculated as follows:

(5) $$\vartheta_{j}=\vartheta_{C}+P_{d}\cdot R_{\vartheta j-C}$$

and:

(6) $$\vartheta_{C}=\vartheta_{S}+P_{d}\cdot R_{\vartheta C-S}$$

which leads to:

(7) $$\begin{array}{c} \vartheta_{j}=\vartheta_{S}+P_{d}\left(R_{\vartheta j-C}+R_{\vartheta C-S}\right) \end{array}$$

where the MOS-FET power dissipation is indicated by $P_{d}$, $R_{\vartheta j-C}$ represents the junction to case thermal resistance for the chosen TO-220 package ($R_{\vartheta j-c}=0.4\;{[}^{o}C/W]$, data-sheet) and $R_{\vartheta C-S}$ is the case to heat sink thermal resistance that must be measured for any particular case, because it depends on different parameters. The case to heat sink resistance ($R_{\vartheta C-S}$) is usually calculated from MOS-FET and heat sink data-sheets, but accurate data are rarely available, especially when isolation (mica) is placed between the MOS-FET case and heat sink. Isolation usually has a significant impact on $R_{\vartheta C-S}$ resistance. It is difficult to mount the temperature sensor on the MOS-FET case, and the easiest solution is to mount it on the heat sink. These values are never completely accurate because of the lack of a model, namely that it does not describe temperature variations over the range of one switching period. Both $R_{\vartheta j-C}$ and $R_{\vartheta C-S}$ values cause uncertainty. It is convenient to perform off-line measurement of this parameter. Figure 5 shows the measurements’ setup.

The MOS-FET was clamped via a spring in the setup device. The transistor was connected to the power supply by appropriate wires. The transistor current was set at approximately 50 A. The MOS-FET case was painted white in order to improve the conditions for the thermal camera measurement. Painted surfaces had a high radiation coefficient (approximately 0.95). The non-painted MOS-FET case has a smaller radiation coefficient, because of case-surface oxidation, and also, its resistance varies widely, causing large measurement errors.

Using the thermal-camera, the temperatures are measured and marked at three points, as indicated in Figure 5. The warmest are MOS-FET terminals (71.2 ${}^{\circ }$C), but the relevant temperatures are the case temperature (53.4 ${}^{\circ }$C) and heat sink temperature (35.9 ${}^{\circ }$C). The thermal measurement results and corresponding calculated values are indicated in Table 3. Based on the measurements, the thermal resistance $R_{\vartheta j-S}$ is evaluated for any particular case. According to the measured current $I_{DS}$ and voltage $U_{DS}$ (Table 3), the MOS-FET $R_{DS-ON}=4.56\;m\text{Ω}$ resistance is calculated, and by rearrangingEquation (3) into:

(8) $$\vartheta_{j}=\frac{-K_{1}+\sqrt{K_{1}^{2}-4K_{0}\left(K_{2}-r\right)}}{2K_{0}}$$

it is possible to evaluate the MOS-FET junction temperature, which is also indicated in Table 3. Finally, the measured (evaluated) thermal resistance $R_{\vartheta j-S}$ is indicated in the last column of thesame Table.

**Table 3:** Temperature characteristics’ coefficients.

*I*	$U_{\mathrm{DS}}$	$\vartheta_{c}$	$\vartheta_{s}$	$P=U_{\mathrm{DS}}\cdot I$	$R_{\mathrm{DS}-\mathrm{ON}}=\frac{U_{\mathrm{DS}}}{I}$	$\vartheta_{j}=f\left(R_{\mathrm{DS}-\mathrm{ON}}\right)$	$R_{\vartheta j-S}$
(A)	(mV)	(${}^{\circ }$C)	(${}^{\circ }$C)	(W)	(m*Ω*)	(${}^{\circ }$C)	(${}^{\circ }$C/W)
45.2	206	53.4	35.9	9.31	4.56	58.6	2.43

### 2.2. Estimation of MOS-FET Power Dissipation

**Figure 6:**
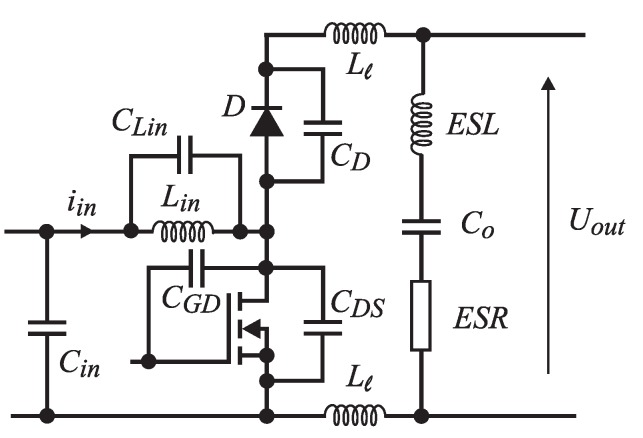
Parasitic elements in the boost converter.

**Figure 7:**
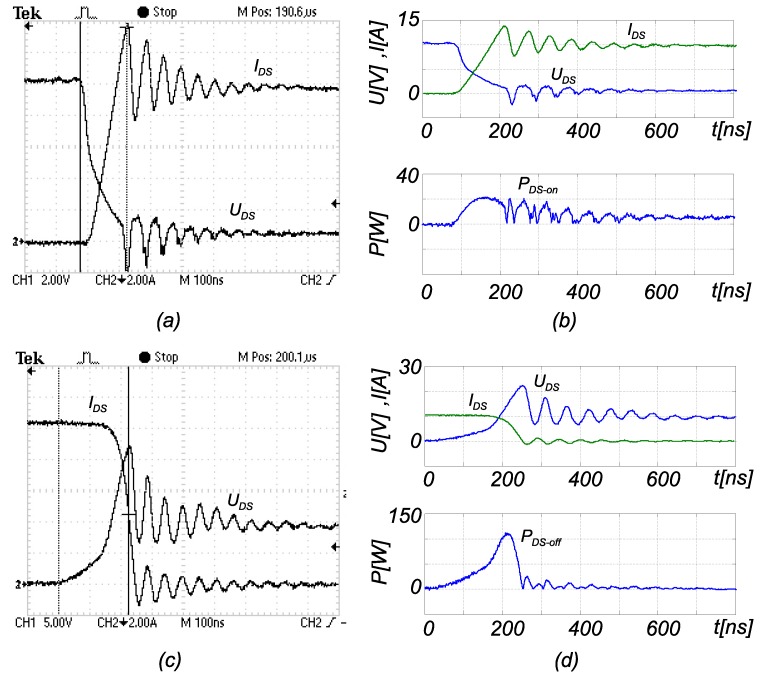
MOS-FET’s turn-on/-off waveforms; (**a**) oscillogram of voltage $U_{DS}$ and current $I_{D}$ during the switching-on; (**b**) oscillogram exported to the MATLAB frame for the calculation of switching on dissipation; (**c**) oscillogram of voltage $U_{DS}$ and current $I_{DS}$ during the switching off; (**d**) oscillogram exported to the MATLAB frame for calculation of switching off dissipation.

**Figure 8:**
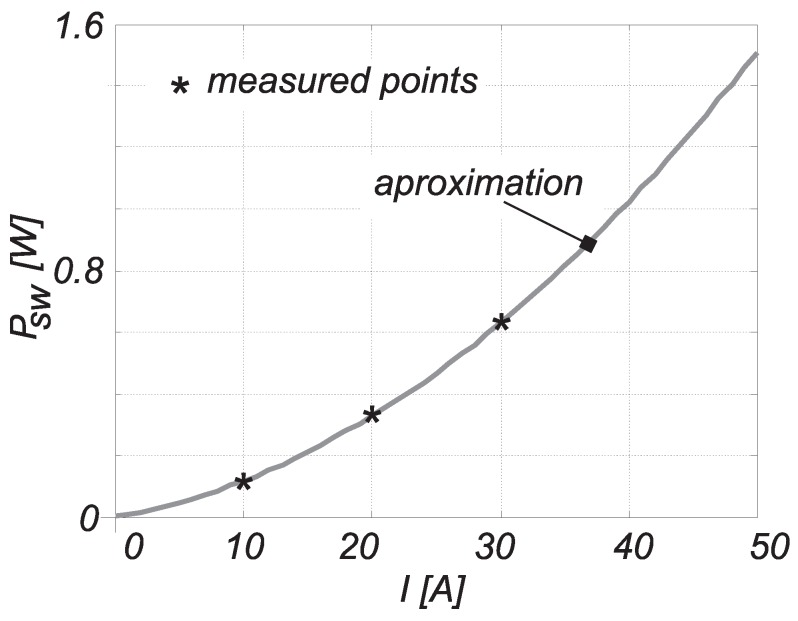
The MOS-FET’s switching losses.

As can be seen from Equation (4), $P_{d}$ contains two parts; the conductive $P_{con}$ and switching $P_{sw}$ losses. Conductive losses are deterministic for small currents and can be calculated as follows:

(9) $$P_{con}=U_{DS}\cdot I_{DS}\cdot \delta$$

where $U_{DS}$ is transistor voltage, when the transistor is conducting the current $I_{DS}$, and *δ* is a duty cycle ratio. At higher currents, the MOS-FET’s terminals and bonding wires also heat-up significantly (they are insulated within plastic housing). The interesting fact is also that the current value $I_{DS}$ is unknown during power dissipation calculation, because the value itself is a result of a measurement algorithm. Therefore, in order to evaluate $P_{con}$, it is necessary to use the current measured over previous switching period, which causes the inaccuracy of the measurement procedure. The second part of the losses are the switching losses, which are even harder to evaluate. Different equations exist, but they do not consider all of the effects within the measurement circuit. In a real circuit, the parasitic inductances and capacitances of tracks and elements cause oscillations in the currents and voltages, and this results in some additional switching losses. Figure 6 shows these inductances and capacitances on the boost converter circuit example. As switching losses calculation is always an approximation, they were measured with an oscilloscope. During MOS-FET turn-on and turn-off, the currents and voltages on the terminals were measured simultaneously. Figure 7 shows waveforms during MOS-FET turn-off at the current of 10 A and supply voltage of 10 V. The waveforms were recorded with a Tektronix oscilloscope, that has a bandwidth of 100 MHz, and the current probe was Tektronix TCP305A with amplifier TCPA300. The probe’s bandwidth is 50 MHz, and the signal propagation delay is 19 ns. The problem also occurs in the $U_{DS}$ voltage measurement, that is inaccurate because of the parasitic inductances of terminals and bonding wires in the transistor. The switching losses were calculated from the acquired waveforms, by using:

(10) $$P_{sw\;ON/OFF}=\frac{1}{T_{s}}\underset{W_{ON/OFF}}{\underset{︸}{\sum_{n=1}^{n_{ON/OFF}}u_{n}\cdot i_{n}\cdot \text{Δ}t_{n}}}$$

where $u_{n}$ and $i_{n}$ are the instantaneously-measured voltage and current values over equidistant time intervals of $\text{Δ}t_{n}$. Index ON/OFF suggests that the equation can be used in turn-on or turn-off cases. The data were obtained from the oscilloscope in the form of a CSV (Comma Separated Value) file and transferred to MATLAB for further processing. The calculation was performed under the MATLAB framework, where the current signal delay had also to be accounted for by moving the waveform left, considering the propagation delay of 19 ns. In Figure 7b,d, the instantaneous losses’ waveforms’ are shown during the switching on and off. The total value of the switching losses was calculated by Equation (10). This procedure was used to measure the turn-on and turn-off losses at three different currents 10,20and30 A, as collected in Table 4. Figure 8 shows the switching losses depending on current $I_{DS}$ ($P_{sw}=P_{sw}\left(I_{DS}\right)$), when the voltage is kept constant at 10 V. It was approximated by a second order polynomial, calculated from the measurement results from Table 4 as follows:

(11) $$P_{sw}=4.6\cdot 10^{-4}I_{DS}^{2}+7.2\cdot 10^{-2}I_{DS}$$

**Table 4:** Calculated switching losses by using Equation (10).

$I_{\mathrm{DS}}$ (A)	$W_{ON}$ (μJ)	$W_{OFF}$ (μJ)	$W_{sw}$ (μJ)	$P_{sw}$ (W)
10	2.06	9.70	11.80	0.118
20	4.13	28.70	32.80	0.328
30	7.11	55.90	63.00	0.630

The constant supply voltage is assumed for power converters. When powered from batteries, the voltage can vary, but within a narrow range.

### 2.3. Bandwidth Evaluation for the $U_{DS}$ Measurement Chain

**Figure 9:**
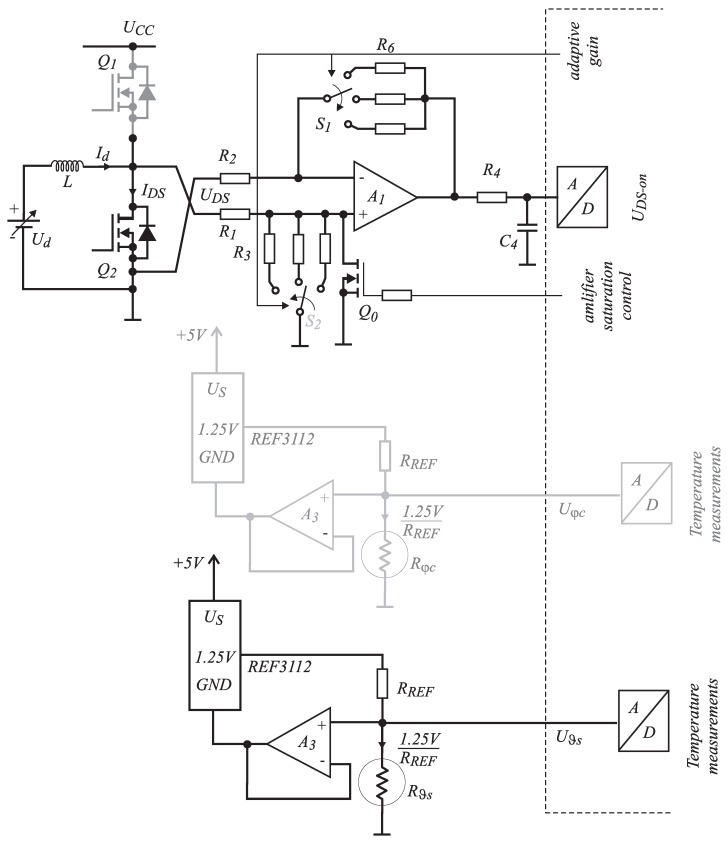
Differential amplifier scheme for $U_{DS}$ measurement and the temperature sensors’ schemes.

**Figure 10:**
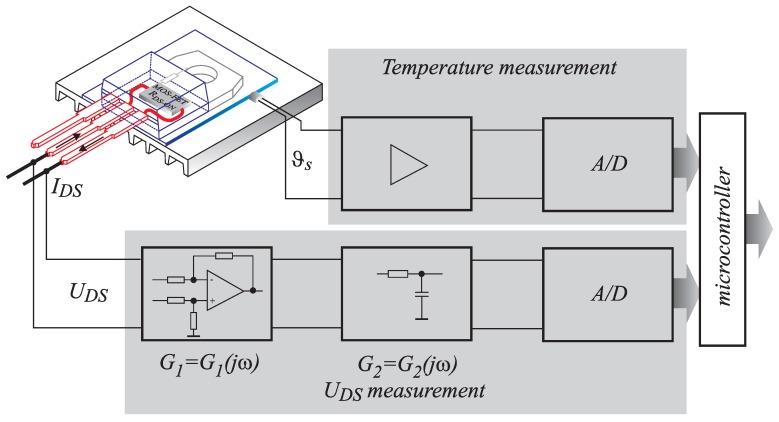
Block scheme of voltage $U_{DS}$ and sink temperature $\vartheta_{s}$ measurement chains.

**Figure 11:**
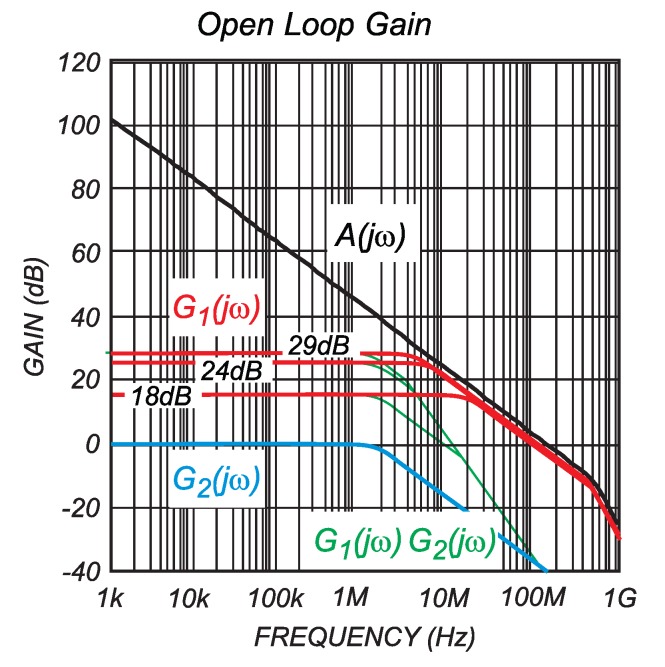
Frequency characteristic of open-loop gain for LMH6611 (black line), closed-loop gains (red lines) and whole measurement chain (tiny green lines).

The bandwidth of the complete measurement chain (Figure 9) needs to be considered. The targeted bandwidth of the method must be at least equal to the one provided by the use of classical magnetic sensors. Figure 10 shows the block scheme of measurement system, which consists of two frequency-dependent analog circuits. The first one represents differential amplifier with open-loop gain characteristic, as shown in Figure 11 [15] (black line), and the second one represents the switching noise filter (blue line). The frequency characteristics can be calculated as presented in [16]. According to the proposed differential amplifier scheme shown in Equation (9), the equation to be applied is:

(12) $$G_{1}\left(j\omega \right)=\frac{R_{6}}{R_{2}}\frac{1}{1+\left(1+\frac{R_{6}}{R_{2}}\right)\frac{1}{A(j\omega )}}$$

where the open-loop gain A(jω) is defined as:

(13) $$A\left(j\omega \right)≅\frac{A_{o}}{1+\frac{j\omega }{\omega_{t}/A_{o}}}$$

and $A_{o}$ is the open-loop gain of amplifier $A_{1}$ (Figure 9) at the frequency ω=0rad/s and has a very high value (infinity in the ideal case) and $\omega_{t}$ represents the cross-over frequency at unity open-loop gain (|A(jω)|=1or0 dB). It must be emphasized that Equation (12) is correct only when $R_{6}/R_{2}=R_{3}/R_{1}$ (Figure 9). By performing an analysis using Equations (12) and (13), the closed-loop frequency characteristic can be evaluated as:

(14) $$G_{1}\left(j\omega \right)≅\frac{G_{o}}{1+\frac{j\omega }{\omega_{b}}}$$

where $\omega_{b}=\omega_{t}/\left(1+R_{6}/R_{2}\right)$ and $G_{o}=R_{6}/R_{2}$ represents the closed-loop gain at the frequency of zero. The quantitative assessments of the bandwidth using Equation (14) for the differential amplifier of different $G_{o}$ (switches $S_{1}$ and $S_{2}$ in Figure 9 can be in three positions) are indicated in Table 5. The three calculated closed-loop frequency characteristics are featured in the Figure 11 (red lines).

**Table 5:** Quantitative assessment of closed-loop differential amplifier gain and bandwidth.

Switches $S_{1}\;\mathrm{and}\;S_{2}$	$R_{6}$	$R_{2}$	$|G_{o}|$ (dB)	Bandwidth (MHz)
Position	kΩ	kΩ	$=20\log(R_{6}/R_{2})$	$f_{b}=\omega_{b}/\left(2\pi \right)$
1	133%±1%	4.8%±1%	29	4.0
2	82%±1%	4.8%±1%	24	6.4
3	39%±1%	4.8%±1%	18	12.4

Further, due to the “hard” switching operation of the observed MOS-FET, the switching noise can disturb the A/D conversion. For this reason, a first order passive filter, which consists of $R_{4}$ and $C_{4}$, was introduced. The frequency characteristics of this filter are given as:

(15) $$G_{2}\left(j\omega \right)≅\frac{1}{1+\frac{j\omega }{\omega_{RC}}}$$

where $\omega_{RC}=R_{4}C_{4}$. According to the chosen values of $R_{4}=200\text{Ω}$ and $C_{4}=390$ pF, the bandwidth of this filter was evaluated as $\omega_{RC}$, ($f_{RC}=\omega_{RC}/\left(2\pi \right)≅2$ MHz). The frequency characteristics of the switching noise filter is also featured in Figure 11 (blue line). The complete transfer function, which can be obtained by multiplying $G_{1}$ and $G_{2}$, is also presented in the Figure 11 by thin green lines. Therefore, it can be concluded that the bandwidth for such an organized shunt-measurement principle is limited to 2 MHz, which is definitely a higher value than the one that can be reached by using the ordinary magnetic principle.

Due to the component tolerances, the response of the actual differential amplifier, which is the crucial part of the system, will deviate from the ideal one. As a means for predicting the extent of this deviation, the sensitivity analysis was performed. The sensitivities can be quantified using the classical sensitivity function $S_{x}^{y}$, defined as:

(16) $$S_{x}^{y}=\frac{\partial y}{\partial x}\frac{x}{y}$$

where *x* denotes the value of component (according to Equation (14), a gain $G_{o}$, a frequency $\omega_{b}$), and *y* denotes the circuit parameter of interest (according to Equation (14), the closed-loop gain in absolute form $|G_{1}\left(j\omega \right)|$). The expression Equation (14) has to be written in its absolute form as follows:

(17) $$G_{A}=\left|G_{1}\left(j\omega \right)\right|=\frac{G_{o}}{\sqrt{1+{\left(\frac{\omega }{\omega_{b}}\right)}^{2}}}$$

Thus, the two sensitivities ($S_{G_{o}}^{G_{A}}$ and $S_{\omega_{b}}^{G_{A}}$) would be evaluated as follows:

(18) $$S_{G_{o}}^{G_{A}}=\frac{dG_{A}}{dG_{o}}\frac{G_{o}}{G_{A}}=1$$

(19) $$S_{\omega_{b}}^{G_{A}}=\frac{dG_{A}}{d\omega_{b}}\frac{\omega_{b}}{G_{A}}=\frac{\omega^{2}}{\omega_{b}^{2}+\omega^{2}}$$

Low-frequency gain $G_{o}$ depends on resistances $R_{6}$ and $R_{2}$; if both have the precision of ±1%, the $G_{o}$ changes for ±2%. Therefore, after using Equation (18), the low-frequency gain changes cause the following:

(20) $$\frac{dG_{A}}{G_{A}}=S_{G_{o}}^{G_{A}}\frac{dG_{o}}{G_{o}}=\pm 2\%$$

A similar analysis can be performed by using Equation (19). This sensitivity is frequency dependent, so two cases will be considered, the first one when the frequency ω=0 and second the one when $\omega =\omega_{b}$. Thereby:

(21) $$S_{\omega_{b}}^{G_{A}}{|}_{\omega =0}=\frac{\omega^{2}}{\omega_{b}^{2}+\omega^{2}}=0$$

and:

(22) $$S_{\omega_{b}}^{G_{A}}{|}_{\omega =\omega_{b}}=\frac{\omega^{2}}{\omega_{b}^{2}+\omega^{2}}=\frac{1}{2}$$

Using the sensitivity evaluated above, the variation of the closed-loop gain $G_{A}$ can be evaluated by:

(23) $$\frac{dG_{A}}{\omega_{b}}{|}_{\omega =0}=S_{\omega_{b}}^{G_{A}}\frac{d\omega_{b}}{\omega_{b}}=0$$

and

(24) $$\frac{dG_{A}}{G_{A}}{|}_{\omega =\omega_{b}}=S_{\omega_{b}}^{G_{A}}\frac{d\omega_{b}}{\omega_{b}}=\pm 0.95\%$$

Thus, by performing a complete sensitivity analysis, it can be concluded that the parameter (resistances) changes cause the tolerance of the closed-loop gain $G_{A}$ to be in the range of ±2%.

### 2.4. $U_{DS}$ Measurement: Anti-Saturation Circuit

As can be concluded from the previous analysis of the MOS-FET ON resistance that it represents a small quantity due to the fact that the voltage $U_{DS}$ (maximum 300 mV) is also of a small value contaminated by switching noise. Figure 9 shows the scheme of the signal acquisition measuring circuit. The noise represents a significant part of a signal, so the usage of a differential amplifier will improve the signal-to-noise ratio. The operational amplifier is saturated when the on-state time of $Q_{1}$ is much longer than the $Q_{2}$. At the sampling time, when $Q_{2}$ is ON for a short time, the amplifier output cannot be set to the correct value (due to the operational amplifier bandwidth), and the sensed voltage value contains an error. In order to cope with the problem, a small MOS-FET $Q_{0}$ was added to the circuit, which is triggered by the same signal as $Q_{1}$. In order to achieve the highest A/D resolution, adaptive gain was introduced by using switches $S_{1}$ and $S_{2}$. The measurements of $U_{DS}$ and temperature $\vartheta_{s}$ are performed as follows from the measurement scheme presented in Figure 9. In order to test the current measurement chain, the current $I_{d}$ (flowing through inductor *L*) was set to 5 A, and the expected measured voltage can be calculated as:

(25) $$U=I_{d}\cdot R_{DS-ON}\cdot G=5\;A\cdot 3.7\;m\text{Ω}\cdot 8.15=150.8\;\mathrm{mV}$$

**Figure 12:**
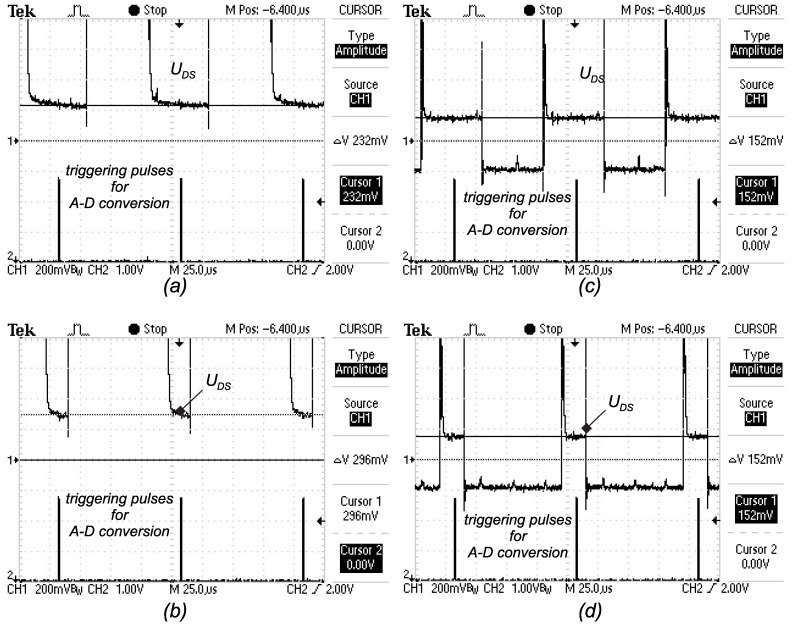
Measurement of $U_{DS}$.

However, during the actual measurement, a voltage of 232 mV was measured, which is different than those calculated and expected (150.8 mV). The low duty cycle on $Q_{2}$ causes the $Q_{1}$ connect voltage $U_{CC}$ on the instrumental operational amplifier ($A_{1}$) input, as indicated in Figure 9, and the amplifier becomes saturated. Due to limited band width, the amplifier output does not reach the steady state in the middle of the $T_{on}$ interval, because $U_{DS}$ goes from saturation to the right value, as shown in Figure 12a,b. Therefore, when A/D was triggered, the $U_{DS}$ voltage was not the right one. Afterwards, the same test was performed when $Q_{0}$ was activated, and the results are presented in Figure 12c,d. The performance index is indicated in Table 6. MOS-FET $Q_{0}$ clearly prevents the amplifier reaching saturation during low duty cycles (when $Q_{1}$ is switched on for a longer time interval).

**Table 6:** $U_{DS-ON}$, instrumental amplifier and anti-saturation effect.

Calculated	Measured	Measured
Voltage (mV)	Voltage (mV) ($Q_{0}$ is OFF)	Voltage (mV) ($Q_{0}$ is ON)
150.8	232.0	152.0

## 3. Experimental Setup

A test circuit was designed for experimental purposes. It also includes the Hall sensor (LEM-HTB100p) in order to verify the proposed algorithm. A program was written for the ARM microcontroller STM32F407, which controls the MOS-FETs and captures $U_{DS}$ and heat sink temperature $\vartheta_{S}$ during circuit operation. The MOS-FET dissipation, junction temperature, transistor current $I_{DS}$ and, consequently, the average value of inductor current $I_{d,av}$ are calculated during every switching period. The switching frequency was 10 kHz. Serial communication makes it possible to transfer the calculated data to a PC and to oversee the circuit operation.

## 4. Performed Measurements, Results and Discussion

The power source supply unit PS-3010 was used for all tests. It is rated for voltages 0–30 V and a current range of 0–10 A. During verification of the $U_{DS}$ measurement amplifier under a small duty cycle (the *δ*-duty cycle was less than 20%), the PS-3010 could provide more than its rated current for a short time (up to 40 A). An inductor of 216 μH was used in the converter circuit. The current ripple ΔI at a switching frequency of 10 kHz was less than 1 A. The MOS-FET current measurement was performed by measuring and calculating in the next steps:

1. synchronized measuring of $U_{DS}$ when the transistor is switched on;
2. measuring of heat sink temperature $\vartheta_{S}$;
3. using the current from the previous measurement to calculate the power dissipation $P_{d}=P_{sw}+P_{con}$, where the switching losses are evaluated using formula $P_{sw}=4.6\cdot 10^{-4}I_{d,av}^{2}+7.2\cdot 10^{-2}I_{d,av}$ and conductive losses are evaluated as $P_{con}=\frac{U_{DS}^{2}}{R_{DS-ON}};$
4. junction temperature is evaluated using $\vartheta_{j}=\vartheta_{S}+P_{d}\left(R_{\vartheta j-C}+R_{\vartheta C-S}\right)$; for initial measurement, there is no previous current $I_{DS}$ data, so $\vartheta_{j}$ is considered equal to $\vartheta_{S}$.
5. based on estimated $\vartheta_{j}$, the $R_{DS-ON}^{*}$ is recalculated;
6. the real current $I_{d,av}^{*}$ is calculated using measured $U_{DS}$ and evaluated $R_{DS-ON}^{*}$.

**Figure 13:**
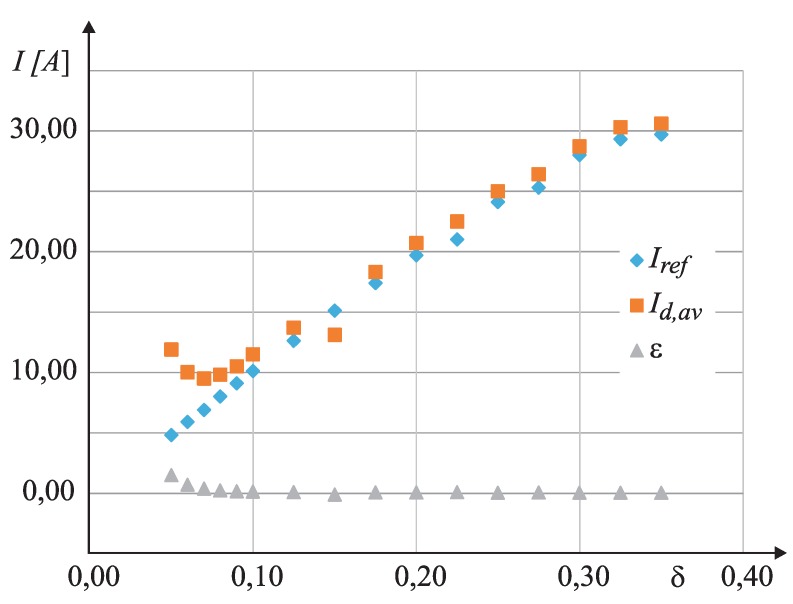
The static characteristics of reference measurement system and the proposed measurement algorithm.

In order to verify the obtained results and for the comparison between the reference measurement system based on the magnetic Hall sensor, the proposed measurement procedure was performed based on the $U_{DS}$ measurement. The duty cycle ($\delta =T_{on}/T$) was modulated from 0.05–0.3. Both measurements were performed; $I_{d,av}$ was measured using the proposed algorithm ($U_{DS}\;\mathrm{and}\;\vartheta_{S}$ measurement) and $I_{ref}$ using the Hall sensor. the obtained results are shown in Table 7. Figure 13 shows the static characteristic of reference and real current measurements. Significant relative error *ε* appears when applying a relatively small duty cycle (0.05<δ<0.1). This is expected, as, due to the relatively low-pass bandwidth of the operational amplifier, the anti-saturation circuit is incapable of setting the output of the instrumental amplifier at the correct value in a very short time [17].

**Table 7:** Verifications of the proposed measurement algorithm.

*δ*	$I_{ref}$	$I_{d,av}$	$\varepsilon =\left(I_{d,\mathrm{av}}-I_{\mathrm{ref}}\right)/I_{\mathrm{ref}}$
0.050	4.8	11.9	1.4792
0.060	5.9	10.0	0.6949
0.070	6.9	9.5	0.3768
0.080	8.0	9.8	0.2250
0.090	9.1	10.5	0.1538
0.100	10.1	11.5	0.1386
0.125	12.6	13.7	0.0873
0.150	15.1	16.1	0.0662
0.175	17.4	18.3	0.0517
0.200	19.7	20.7	0.0507
225	21.0	22.5	0.0571
0.250	24.1	25	0.0373
0.275	25.3	26.4	0.0435
0.300	28.0	28.7	0.0250
0.325	29.3	30.3	0.0341
0.350	29.7	30.6	0.0303

**Figure 14:**
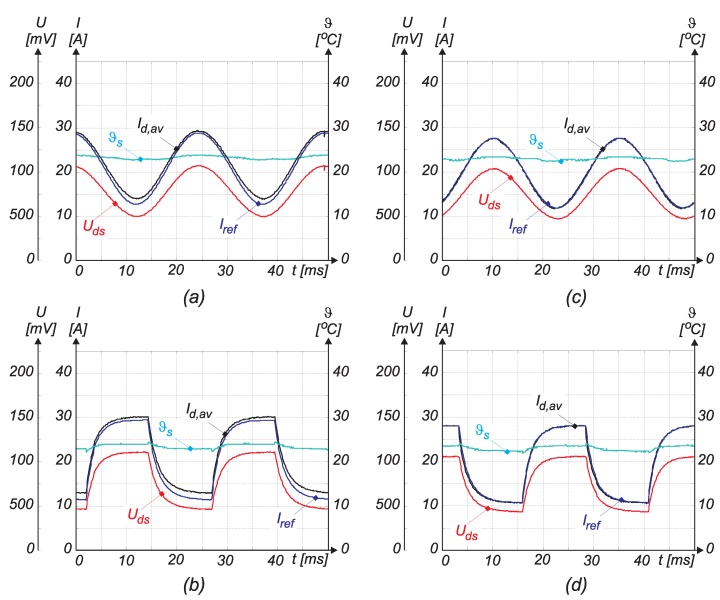
Sine wave and square modulation when δ∈ ( 0.1–0.3); (a) measured $U_{DS}$, $\vartheta_{s}$, $I_{ref}$ and calculated $I_{d,av}$ without compensation (sinus modulation); (b) measured $U_{DS}$, $\vartheta_{s}$, $I_{ref}$ and calculated $I_{d,av}$ without compensation (square modulation); (c) measured $U_{DS}$, $\vartheta_{s}$, $I_{ref}$ and calculated $I_{d,av}$ with compensation (sinus modulation); (d) measured $U_{DS}$, $\vartheta_{s}$, $I_{ref}$ and calculated $I_{d,av}$ with compensation (square modulation).

Furthermore, the proposed algorithm was also tested for the sine wave and square modulation (0.1<δ<0.3). The raw measurement results were acquired in real time by a microcomputer and then processed in MATLAB. The maximal errors ($I_{d,av}$ compared with $I_{ref}$) were between 10.2% and 13.2% and are extracted, respectively, from Figure 14a,b, where the obtained results are presented. The voltage $U_{DS}$ and heat sink temperature are also acquired and displayed in Figure 14a,b. As was expected from previous verification of the proposed algorithm, the error appeared because of the poor performance of the sensor at low duty cycles. The acquisition circuit is not fast enough to track $U_{DS}$ during the short conduction time of $Q_{2}$. Calibration based on the reference current measurement by the Hall sensor was performed in order to compensate for this error. It was measured for some small values of the duty cycle. The static characteristic $\varepsilon =\varepsilon \left(I_{d,av},I_{ref}\right)$ was approximated by the function:

(26) $$\varepsilon \left(\delta \right)=\frac{I_{d,av}-I_{ref}}{I_{ref}}=\frac{a}{{\left(\delta -b\right)}^{2}}+c$$

where coefficients *a*, *b* and *c* are indicated in Table 8. The order of the function and coefficients was determined experimentally. By the help of Equation (26), the measured results were compensated. The performed algorithm is calibrated with the reference current measurement. The obtained results are shown in Figure 14c,d. The performance index without and with compensating approaches is indicated in Table 9. The voltage $U_{DS}$ and heat sink temperature are also acquired and displayed in Figure 14c,d.

**Table 8:** Error characteristic’s coefficients.

Coefficients	*a*	*b*	*c*
–	580	30	0.02

**Table 9:** Error characteristics’ coefficients.

Coefficients	Error without Compensation	Error with Compensation
sine wave	10.2%	<2%
square wave	13.2%	<2%

## 5. Conclusions

Based on the obtained results, it is possible to claim that MOS-FETs can be used as sensors in power electronics’ converters. Clean signals can be achieved, with careful measurement circuit design, which are comparable to other sensor types. This sensor has many advantages; the measurement principle does not add many additional losses to the converter circuit, thus eliminating the offset problem of the Hall sensors. The main obstacle for this measurement principle is the changes of $R_{DS-ON}$ during the operation at small duty cycles and due to the junction temperature. The temperature dependence of $R_{DS-ON}$ must be considered. Approximation with potential function was proposed for limited accuracy at small duty cycles. As a testing platform, the step-up converter was chosen, and the obtained results are promising.

## References

[1] Wrzecionko B., Steinmann L., Kolar J.W. (2013). High-bandwidth high-temperature (250 °C/500 °F) isolated DC and AC current measurement: Bidirectionally saturated current transformer. IEEE Trans. Power Electron..

[2] Lee W.-C., Hyun D.-S., Lee T.-K. (2000). Novel control method for threephase PWM rectifiers using a single current sensor. IEEE Trans. Power Electron..

[3] Zidat F., Lecointe J., Morganti F., Brudny J., Jacq T., Streiff F. (2010). Non invasive sensors for monitoring the efficiency of AC electrical rotating machines. Sensors.

[4] Reyes Barranca M., Mendoza-Acevedo S., Flores-Nava L., Avila-Garcia A., Vazquez-Acosta E., Moreno-Cadenas J., Casados-Cruz G. (2010). Using a floating-gate MOS transistor as a transducer in a MEMS gas sensing system. Sensors.

[5] Dai C., Tai Y., Kao P. (2007). Modeling and fabrication of micro FET pressure sensor with circuits. Sensors.

[6] Dai C., Lu P., Wu C., Chang C. (2009). Fabrication of wireless micro pressure sensor using the CMOS process. Sensors.

[7] Kim H.R., Jahns T.M. (2006). Phase current reconstruction for ac motor drives using a dc-link single current sensor and measurement voltage vectors. IEEE Trans. Power Electron..

[8] Cusido J., Romeral L., Ortega J., Garcia A., Riba J. (2011). Signal injection as a fault detection technique. Sensors.

[9] Aiello O., Fiori F. (2013). A new mirroring circuit for power MOS current sensing highly immune to EMI. Sensors.

[10] Lavrič H., Fišer R. (2010). Lossless current sensing technique on MOSFET RDS(on) with improved accuracy. Electronics Letters.

[11] Chen H., Pickert V., Atkinson D.J., Pritchard L.S. On-line monitoring of the MOS-FET device junction temperature by computation of the threshold voltage. Proceedings of the 3rd IET International Conference Power Electronics, Machines and Drives (PEMD 2006).

[12] Musallam M., Acarnley P.P., Johnson M., Pritchard L. Real-time power electronic device junction temperature estimation. Proceedings of the Second International Conference on Power Electronics, Machines and Drives (PEMD 2004).

[13] (2009). International Rectifier, IRFB4110GPbF: HEXFET Power MOS-FET. http://www.irf.com/product-info/datasheets/data/irfb4110pbf.pdf.

[14] Buttay C., Bergogne D., Herve M., Bruno A., Rene E., Pascal B. Towards a Sensorless Current and Temperature Monitoring in MOS-FET-Based H-Bridge. Proceedings of the 2003 IEEE 34th Annual Power Electronics Specialist Conference.

[15] (2013). Texas Instruments, LMH6611: Single Supply 345 MHz Rail-to-Rail Output Amplifiers. http://www.ti.com/lit/ds/symlink/lmh6611.pdf.

[16] Sedra A.S., Smith K.C. (2009). Microelectronic Circuits.

[17] Pajer R. (2014). Current Sensing on Power MOS-FET in Electrical Motor Drives. Masters Thesis.

